# Multi-modal spatial glycomic analysis of lymphoid aggregates in prostate and pancreatic cancer tissues

**DOI:** 10.3389/fonc.2026.1935795

**Published:** 2026-09-29

**Authors:** Kameisha V. Rashford, Caroline G. Kittrell, Lyndsay E. A. Young, Dessie Anne Nietert, Colin T. McDowell, Grace Grimsley, Blake E. Sells, Kaitlyn Bejar, Anand S. Mehta, Liang-I Kang, Peggi M. Angel, Jennifer D. Wu, David G. DeNardo, Robin J. Leach, Joseph E. Ippolito, Richard R. Drake

**Affiliations:** 1 Department of Pharmacology and Immunology and The Hollings Cancer Center, Medical University of South Carolina, Charleston, SC, United States; 2 Division of Oncology, Washington University in Saint Louis School of Medicine, Saint Louis, MO, United States; 3 Department of Cell Systems and Anatomy, University of Texas Health Science Center, San Antonio, TX, United States; 4 Department of Pathology and Immunology, Washington University in Saint Louis School of Medicine, Saint Louis, MO, United States; 5 Department of Urology, Feinberg School of Medicine, Northwestern University, Chicago, IL, United States; 6 Department of Radiology, Washington University in Saint Louis School of Medicine, Saint Louis, MO, United States

**Keywords:** lymphoid aggregates, MALDI-IHC, MALDI-MSI imaging, N-glycosylation, pancreatic adenocarcinoma, prostate adenocarcinoma, spatial biology, tumor microenvironment (TME)

## Abstract

**Introduction:**

Altered N-glycosylation plays a critical role in immune regulation, yet the spatial distribution of N-glycans within immune niches of immunologically “cold” tumors remain poorly defined. Here, we combine matrix-assisted laser desorption/ionization mass spectrometry imaging (MALDI-MSI) with highly multiplexed immunohistochemistry using photocleavable mass tags. This integrated workflow enables spatial mapping of N-glycosylation and immune markers within lymphoid aggregates (LAs) in prostate and pancreatic ductal adenocarcinoma tissues, two malignancies that respond poorly to immunotherapy. Given the role of N-glycosylation in immune regulation, spatially resolved N-glycan profiling may provide insight into molecular heterogeneity within lymphoid aggregates and their surrounding tumor microenvironment.

**Methods:**

To assess the relationship between N_glycosylation and immune markers, candidate LAs from tissue samples collected from patients with varying stages of prostate cancer, PCa (n=49) and pancreatic ductal adenocarcinoma, PDAC (n=26) were identified via histological stains prior to enzymatic release of N-glycans and MALDI-MSI. MALDI-IHC was used to target immune cell markers and extracellular matrix proteins.

**Results:**

In tissues with LAs, three N-glycan structural classes were identified when detectable: oligo-mannose, fucosylated tetra-antennary, and bisecting N-acetylglucosamine in fucosylated bi,- tri- and tetra- antennary structures, with some N-glycans common to both tumor types. LAs showed predominantly central CD20 and CD44 signal surrounded by CD3 signal localized along the tumor periphery.

**Conclusion:**

Our analysis revealed unique N-glycan patterns within and surrounding LAs, with variable profiles in distant regions, highlighting heterogeneity in LA-associated N-glycan profiles.

## Introduction

Although prostate cancer (PCa) and pancreatic ductal adenocarcinoma (PDAC) are distinct in their respective pathophysiologies, average survival times, and therapeutic regimens (1, 2), both tumors are characterized by resistance to treatment with immunotherapy, specifically immune checkpoint inhibition (ICI) (3–8). While ICI has been effective in many hematologic malignancies (9) and some solid tumors such as melanoma (10), lung (11), triple-negative breast cancer (12), and renal cell carcinoma (13), it has shown limited success in PCa and PDAC. At advanced stages, these cancers have a poor 5-year survival rate and primarily rely on non-immunotherapy systemic treatments (14). Immunotherapy is generally more effective in tumors with high immune cell infiltration, like lung and melanoma, while those with low infiltration tend to respond poorly. However, some highly infiltrated tumors still show strong resistance to treatment (15–17). This has led researchers to investigate the presence of immune cells, their organization, and spatial relationships within the surrounding tumor microenvironment (TME) (18). Of particular interest are tertiary lymphoid structures (TLS), which are ectopic lymphoid aggregates (LAs) that forms in non-lymphoid tissues during chronic inflammation (19–24). TLS are typically characterized by a core of CD20^+^ B cells embedded within a network of T lymphocytes, stromal cells, dendritic cells, and associated extracellular matrix components (21, 25, 26). TLS are formed through a step-wise progression from immature LAs, to primary follicular-like structure with B cell and T cell zonation, and ultimately to mature TLS with a germinal center (21). Because the histologically identified structures in this study were not fully classified across these maturation stages, they are referred to here as LAs. Despite increasing evidence that LAs are associated with better prognosis in metastatic colorectal (27, 28), lung (29), and breast (20) cancers, their roles in human PCa and PDAC tissues are still being defined (19).

N-glycosylation, which occurs co-translationally with the covalent attachment of glycans to asparagine residues on proteins, is one of many molecular regulatory processes of immune activity (30). This post-translational modification plays a pivotal role in protein folding, stability, and receptor function (31, 32), and it is also a major component of immune cell recognition and their binding to target cells via a large family of carbohydrate-binding lectins (30, 33). In cancer, alterations in cell surface N-glycosylation are typified by structurally larger and complex glycans that can mask tumor-associated antigens from immune surveillance or engage inhibitory glycan-binding receptors, such as siglecs (34, 35) and galectins (36) on immune cells. Although glycans are recognized as critical regulators of immune function (30), no prior study has simultaneously mapped the spatial distribution of N-glycans with immune cell organization in the TME in PCa and PDAC.

N-glycan mass spectrometry imaging (MSI) is an approach to interrogate and define the spatial distribution of N-glycans in tissues, and it has been applied to multiple tumor types including PCa (37, 38), PDAC (39) and tissue atlas of 15 tumor types (40). Recent advances in multimodal imaging technologies like MALDI-IHC (41, 42) and MIBI-TOF (43) have expanded the utility of N-glycan MSI, making it compatible with complementary techniques that enhance spatial and molecular resolution. Prior studies from our group characterized immune cell glycosylation in various pathological contexts in a few samples, including COVID-19-infected lungs (44), colorectal cancer (45), and renal cell carcinoma (46), as well as a new single-cell platform ideal for immune cells (47). In this study, we used a multi-modal approach including histological stains, MALDI-IHC and N-glycan MSI to characterize LAs in human PCa and PDAC tissues. Histological stains were used to screen tissues for LAs, and MALDI-IHC was used to identify immune cell markers. N-glycan MSI was then used to identify the levels of LA-specific N-glycan signatures for each tissue.

## Methods

### Chemicals and reagents

Miralys™ probes were obtained from Ambergen Inc (Billerica, MA). HPLC-grade water, xylene, citraconic anhydride, and acetonitrile (ACN) were obtained from Fisher Scientific (Hampton, NH). Ethanol was obtained from Decon Labs (King of Prussia, PA). Trifluoroacetic acid (TFA) and α-cyano-4-hydroxycinnamic acid (CHCA) were purchased from Sigma-Aldrich (St. Louis, MO). Peptide-N-glycosidase F (PNGase F) PRIME™ was obtained from N-Zyme Scientific (Doylestown, PA). H&E stains were obtained from Cancer Diagnostics (Durham, NC).

### Clinical tissues

Archived prostate cancer and pancreatic cancer formalin-fixed paraffin embedded (FFPE) tissue samples were obtained from the following institutions under approved IRB protocols: Hollings Cancer Center Biorepository and Tissue Analysis Shared Resource at the Medical University of South Carolina (Charleston, SC); Cancer Tissue Bank and Database at the University of Texas Health Science Center (San Antonio, TX IRB 20050234HR); Northwestern University (Chicago, IL); Washington University School of Medicine (St. Louis, MO IRB 201108117). All samples were obtained without identifiers and could therefore be distributed without an independent IRB protocol. The evaluated tissue set included PCa (n = 49) and PDAC (n = 26). Clinicopathologic information is summarized for the subset of LA-containing tissues included in downstream LA-associated N-glycan analysis: PCa, n = 14 of 49, and PDAC, n = 17 of 26 (**Supplementary Tables 1**, **2**). The PCa cohort primarily included patients with localized to locally advanced disease, predominantly clinical stages T2–T3.Pretreatment serum prostate-specific antigen (PSA) values, body mass index, and demographic data were available for a subset of patients. The PDAC cohort included both male and female patients aged 24–84 years, with tumors that were moderately to poorly differentiated and most frequently presented at T3 stage with regional lymph node involvement. Clinical data, including demographic, disease-specific, and outcome-related variables, were available for most samples; however, a subset of tissue specimens had no associated clinical information. This limits clinical interpretation, as measurements were performed within tissue sections and focused on LAs and N-glycan expression patterns rather than clinical associations.

### MALDI-MSI N-glycan imaging

A standardized protocol was used for tissue preparation, enzymatic digestion, and matrix application as previously described (48). FFPE tissue sections were deparaffinized at 60 °C for 1 hour followed by sequential washes in xylene and graded ethanol solutions, and rehydration in HPLC water. Antigen retrieval was then performed using a citraconic anhydride buffer (25 µL citraconic anhydride, 2 µL 12 M HCl, and 50 mL HPLC-grade water; pH 3.0 ± 0.5) for 30 min at approximately 95 °C using a vegetable steamer. Following antigen retrieval, PNGaseF PRIME (0.1 µg/µL) was applied in 15 passes using an M5™ Sprayer (HTX Technologies LLC, Chapel Hill, NC) at a flow rate of 25 µL/min, with a 3 mm offset, velocity of 1200 mm/min, 45 °C temperature, and 10 psi nitrogen pressure. Next, slides were incubated in pre-heated 37 °C humidified chambers for 2 hours. α-cyano-4-hydroxycinnamic acid (CHCA) matrix (7 mg/mL in 50%ACN/0.1%TFA) was applied using a M5™ Sprayer in 10 passes at 0.1 mL/min, 2.5 mm offset, 1300 mm/min spray velocity, 79 °C, and 10 psi. Following matrix application, slides were desiccated and analyzed using timsTOF Flex MALDI-QTOF mass spectrometer (Bruker Corporation, Billerica, MA). This instrument utilizes a 3D smartBeam laser with a laser spot size of 20µm. Analysis was performed in positive ion mode with 300 shots per pixel over a mass range of 700–4000 m/z with a 40 µm raster. Using SCiLS Lab software v2025a, N-glycan peaks were manually assigned by matching observed masses to theoretical values within ±10 ppm using an in-house database of known N-glycans derived from prior studies 37-40, including features detected in PCa (n = 62) and PDAC (n = 98), and previously reported MS/MS collision-induced dissociation profiles (49, 50).

### MALDI-IHC

Miralys™ probes were used to detect immune cell surface markers and extracellular matrix (ECM) components. FFPE tissue sections were dewaxed by incubation at 60 °C for 2 h, followed by sequential washes in xylene and graded EtOH solutions. Sections were rehydrated in HPLC-grade water and subjected to antigen retrieval using an alkaline ethylenediaminetetraacetic acid (EDTA) buffer (pH 8.5) for 30 min in a vegetable steamer maintained at approximately 95 °C. Following antigen retrieval, tissues were allowed to cool to room temperature and dried in a desiccator. Sections were then incubated for 1h at RT in tissue blocking buffer consisting of 2% rabbit serum, 2% mouse serum, and 5% bovine serum albumin (BSA) prepared in Tris-buffered saline (TBS) supplemented with 0.05% octyl β-D-glucopyranoside (OBG). The probe mixture was applied under light-protected conditions; slides were immediately foil-wrapped and incubated overnight at 4 °C with continuous gentle rocking to promote even probe distribution. After incubation, slides were washed in TBS (3 × 5 min), briefly rinsed in freshly prepared ammonium bicarbonate (NH_4_HCO_3_) for 10s, and further washed in fresh NH_4_HCO_3_ solutions (3 × 2 min). Slides were then dried for 1 h 30 min at RT in a desiccated environment. Photocleavable mass tags were released by exposure to ultraviolet (UV) light for 10 min using an AmberGen light box operated according to manufacturer-recommended settings. CHCA matrix (10 mg/mL in 70% ACN and 0.1% TFA was applied using a TM Sprayer in eight passes with a 100 µL/min flow rate, 1350 mm/min velocity, 60 °C nozzle temperature, 3 mm track offset, and 10 psi nitrogen. Slides were recrystallized using 5% IPA in a 60 °C oven for 1–2 min prior to analysis on a timsTOF Flex MALDI-QTOF mass spectrometer (Bruker Corporation, Billerica, MA). IHC probe analyses were conducted in positive ion mode using 300 laser shots per pixel over a mass range of 800–2000 with a 40-µm raster. Miralys™ probe-associated mass tag peaks were assigned based on manufacturer-provided m/z values from AmberGen Inc.

### Data analysis

Tissue sections were first screened histologically to identify LAs. Sections were stained with Lewis Y, Mayer’s hematoxylin, or hematoxylin and eosin (H&E) and imaged using a NanoZoomer 2.0-RS scanner (Hamamatsu) to confirm the presence of lymphoid cells. MALDI-MSI and MALDI-IHC datasets were co-registered to corresponding histological images using SCiLS Lab software v2025a (Bruker Daltonics). N-glycan and IHC probe ion images were imported independently, spatially aligned, and normalized by total ion current (TIC)(ICR noise reduction threshold 0.95). Relative N-glycan abundances were calculated from total ion–normalized peak lists using maximum peak intensity. Mean MALDI-IHC marker intensities across all LAs per tissue were calculated using Excel and GraphPad Prism. Distances between LAs and adjacent tumor regions were measured in NDPI software, and log_10_-transformed glycan intensities were plotted relative to tumor proximity.

### Co-localization and frequency analysis

For tissues with detectable N-glycans in LAs, co-localization analysis was performed using the “Find Values Co-Localized to Region” workflow in SCiLS Lab v2025a (Bruker) software. Inclusion required well-defined LAs measuring ≥40 µm in diameter with detectable N-glycan signal above background within annotated LA regions. Based on these criteria, PCa (n = 14 of 49) and PDAC (n = 17 of 26) tissues were included for LA-associated N-glycan analysis. This analysis was performed independently for each tissue section and did not involve pooling data across samples. For each tissue, Pearson correlation coefficients (r) were calculated to assess associations between pixel-wise N-glycan signal intensities and spatial inclusion within user-defined LA ROIs. Positive correlation values indicated N-glycan enrichment within the ROI, whereas negative values indicated relative exclusion. Because Pearson correlation compared spatially restricted LA regions against whole-section ion images, correlation coefficients were generally low and were therefore used as a within-tissue ranking metric rather than as a measure of strong linear association across samples. For each tissue section, the top 25 ranked N-glycans with the highest Pearson correlation coefficients were selected. A summary table was generated to organize glycans into mutually exclusive structural categories based on antennarity, bisecting GlcNAc status, and fucosylation. Within each tissue, the median Pearson correlation coefficient (r) was calculated for all glycans in each category. Tissue-level medians were summarized separately for PCa and PDAC using the median and interquartile range (Q1–Q3). For each structural category, we also report the number of tissues with a positive median correlation (r > 0) relative to the number of tissues in which that category was detected (n/N). The frequency of recurring top-ranked N-glycans was then calculated by tallying how often each positively correlated N-glycan appeared among the top 25 ranked features across PCa and PDAC tissues, and the 15 most frequent N-glycans were retained for cohort-level visualization.

## Results

Recent studies from our group have demonstrated the ability to detect N-glycan signatures within intratumor lymphocyte aggregates (LAs) in a few representative colon and kidney cancer FFPE tissues using a combination of N-glycan mass spectrometry imaging (MSI) and MALDI-immunohistochemistry (MALDI-IHC) (45, 46). In the present study, the goal was to systematically identify overall LA N=glycosylation patterns and their frequency of detection in a larger cohort of FFPE prostate cancer (PCa, n=49) and pancreatic cancer (PDAC, n=26) tissues. Tissue sections were screened for histologically identifiable LAs and identified by tissue-based markers and digital pathology tools. All tissues were analyzed by N-glycan MSI, with N-glycans co-localized to known tumor and stroma regions as previously described. For each tissue, the regions with identified LAs were highlighted for co-localization with N-glycan signals. Of the screened tissues, PCa (n = 14 of 49) and PDAC (n = 17 of 26) met inclusion criteria and were included in downstream LA-associated N-glycan analysis (**Supplementary Figure 1**). Their clinical characteristics are summarized in **Supplementary Tables 1** and **2**. Examples of PCa and PDAC tissues containing N-glycan signal within LAs are shown in **Figure 1**. Across both tumor types, LAs were identified in distal and peritumoral regions, with representative histologically stained tissue sections shown in **Supplementary Figure 2**. Many LAs in PDAC were surrounded by whorls of pale staining stroma (**Supplementary Figures 2B, C**). A lymph node with common bile duct metastasis was utilized as a positive control for comparison of staining patterns and typical appearance of normal adjacent lymphoid follicles. The width of LAs were measured using NDPI imaging software. Compared to the positive control lymph node (103–955 µm) (**Supplementary Figure 2C**), LAs in PCa ranged from small aggregates (~40 µm width) to large, well-organized structures (~800 µm width) (**Supplementary Figure 2A**), whereas in PDAC they were generally smaller (91–404 µm) (**Supplementary Figure 2B**).The overall shape of the LAs were used to define co-localized N-glycan species, and there was no association with N-glycan signal and the relative sizes of the LA in both tissue types; i.e., all sizes had potential for N-glycan detection. Representative N-glycan species detected within LA in both PCa and PDAC fell into three major structural groups: 1. oligo-mannose compositions; 2. fucosylated tetra-antennary with or without N-acetylglucosamine additions; 3. bisecting N-acetylglucosamine in fucosylated bi-, tri- and tetra-antennary compositions. These structural groups are summarized in **Figures 1A–F** and **Supplementary Table 3**. An example of PCa and PDAC tissues that did not have co-localized N-glycan signals in their LAs are shown in **Supplementary Figure 3**. It should be noted that there were no apparent differences in the types and levels of N-glycans present in the tumor and stroma regions of these tissues, only the lack of signal in their LAs (**Supplementary Figure 3**).

**Figure 1:**
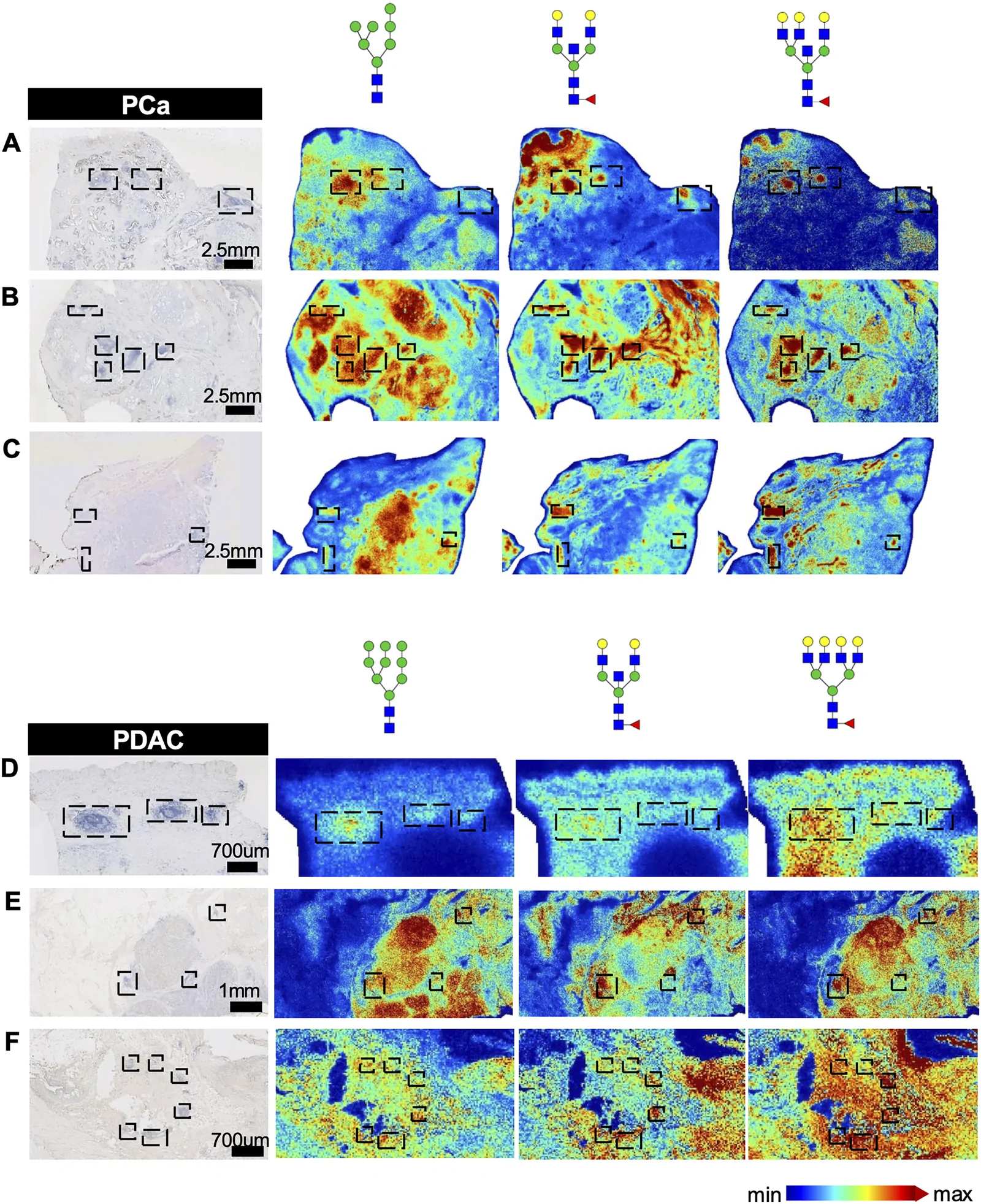
N-glycan signatures within and adjacent to lymphoid aggregates in prostate cancer and pancreatic ductal adenocarcinoma. Mayer’s hematoxylin staining and N-glycan imaging of lymphoid aggregate (LA) regions in **(A)** PCa13, **(B)** PCa6, **(C)** PCa8, **(D)** PDAC15, **(E)** PDAC2, and **(F)** PDAC16, highlighting representative N-glycan species detected across both tumor types.

N-glycans were detected in 85 LA ROI from PCa (n=14) and 78 LA ROI from PDAC tissues (n=17). Co-localization analysis identified N-glycans with positive spatial correspondence to LA-defined regions. For each tissue section, N-glycans were ranked by Pearson correlation coefficient, and the top 25 features were visualized using scatterplots (**Supplementary Figure 4**). To descriptively summarize structural trends among the top 25 LA-associated N-glycans, Pearson correlations were grouped by structural category and analyzed separately for PCa and PDAC (**Table 1**). In PCa, median tissue-level correlations were positive across nearly all structural categories (median r 0.0059 to 0.0468); the only negative value occurred for non-fucosylated, bisected tri-antennary glycans (−0.0235), a category represented by a single tissue. In PDAC, median correlations were positive for every structural category (median r 0.0149 to 0.0702). Correlations were weak in absolute terms in both cancers, and positive associations were not restricted to particular structural categories (**Table 1**; **Supplementary Figure 4**). To identify frequent LA-associated N-glycans within each tumor type, we tallied the occurrence of each glycan among the top 25 positively correlated features. Frequently recurring N-glycans included m/z 2743.978, 2012.687, and 2377.801 in PCa, and m/z 2540.903, 1905.624, 2067.675, and 2377.857 in PDAC (**Figures 2A, B**). Representative ion images of the top positively correlated N-glycan in each cancer are shown in **Figures 2C, D**: m/z 2743.978, a fucosylated, bisected tetra-antennary structure, in PCa6 (r = 0.337), and m/z 2540.903, a fucosylated tetra-antennary structure, in PDAC4 (r = 0.410); corresponding scatterplots are shown in **Supplementary Figure 4**. Additional examples of N-glycan spatial distributions are provided in **Supplementary Figures 5**–**7** for PCa and PDAC, including an overall segmentation analysis of all N-glycans (**Supplementary Figure 5**) and representative ROI images for PCa6 (**Supplementary Figure 6**) and PDAC16 tissues (**Supplementary Figure 7**).

**Table 1:** Summary of LA-associated N-glycan structural categories in PCa and PDAC.

Cancer type	Structural category	Tissues with positive median r, n/N	Median tissue r (Q1–Q3)
PCa	Paucimannose	4/6	0.0244 (-0.0060–0.0480)
	High-mannose	11/14	0.0229 (0.0080–0.0743)
	Hybrid, non-fucosylated	6/8	0.0152 (0.0012–0.0398)
	Hybrid, fucosylated	2/2	0.0255 (0.0254–0.0256)
	Bi-antennary, non-bisected, non-fucosylated	6/9	0.0059 (-0.0088–0.0241)
	Bi-antennary, non-bisected, fucosylated	7/7	0.0283 (0.0161–0.0492)
	Bi-antennary, bisected, non-fucosylated	9/10	0.0468 (0.0268–0.0760)
	Bi-antennary, bisected, fucosylated	11/14	0.0336 (0.0046–0.0774)
	Tri-antennary, non-bisected, non-fucosylated	4/4	0.0353 (0.0177–0.0531)
	Tri-antennary, non-bisected, fucosylated	9/11	0.0274 (0.0099–0.0387)
	Tri-antennary, bisected, non-fucosylated	0/1	-0.0235 (-0.0235–-0.0235)
	Tri-antennary, bisected, fucosylated	12/14	0.0263 (0.0077–0.0455)
	Tetra-antennary, non-bisected, non-fucosylated	5/8	0.0091 (-0.0041–0.0298)
	Tetra-antennary, non-bisected, fucosylated	11/14	0.0223 (0.0068–0.0273)
	Tetra-antennary, bisected, fucosylated	14/14	0.0308 (0.0176–0.0821)
PDAC	Paucimannose	5/6	0.0620 (0.0563–0.0720)
	High-mannose	14/15	0.0392 (0.0292–0.0735)
	Hybrid, non-fucosylated	9/11	0.0416 (0.0155–0.0617)
	Hybrid, fucosylated	5/5	0.0702 (0.0649–0.1033)
	Bi-antennary, non-bisected, non-fucosylated	10/11	0.0501 (0.0404–0.0896)
	Bi-antennary, non-bisected, fucosylated	13/14	0.0390 (0.0240–0.0748)
	Bi-antennary, bisected, non-fucosylated	8/10	0.0441 (0.0118–0.0815)
	Bi-antennary, bisected, fucosylated	12/15	0.0425 (0.0185–0.0860)
	Tri-antennary, non-bisected, non-fucosylated	11/13	0.0475 (0.0307–0.0583)
	Tri-antennary, non-bisected, fucosylated	13/15	0.0465 (0.0305–0.0691)
	Tri-antennary, bisected, non-fucosylated	3/6	0.0149 (-0.0077–0.0317)
	Tri-antennary, bisected, fucosylated	12/12	0.0582 (0.0314–0.1100)
	Tetra-antennary, non-bisected, non-fucosylated	12/12	0.0484 (0.0341–0.0785)
	Tetra-antennary, non-bisected, fucosylated	13/16	0.0520 (0.0236–0.0736)
	Tetra-antennary, bisected, non-fucosylated	3/3	0.0354 (0.0221–0.0406)
	Tetra-antennary, bisected, fucosylated	5/8	0.0357 (-0.0070–0.0421)

*Values are summarized across tissues. n/N denotes the number of included tissues with a positive median Pearson correlation (r > 0) out of the total number of tissues included for that structure category. Q1–Q3 represents the interquartile range of tissue-level median Pearson r values.*

**Figure 2:**
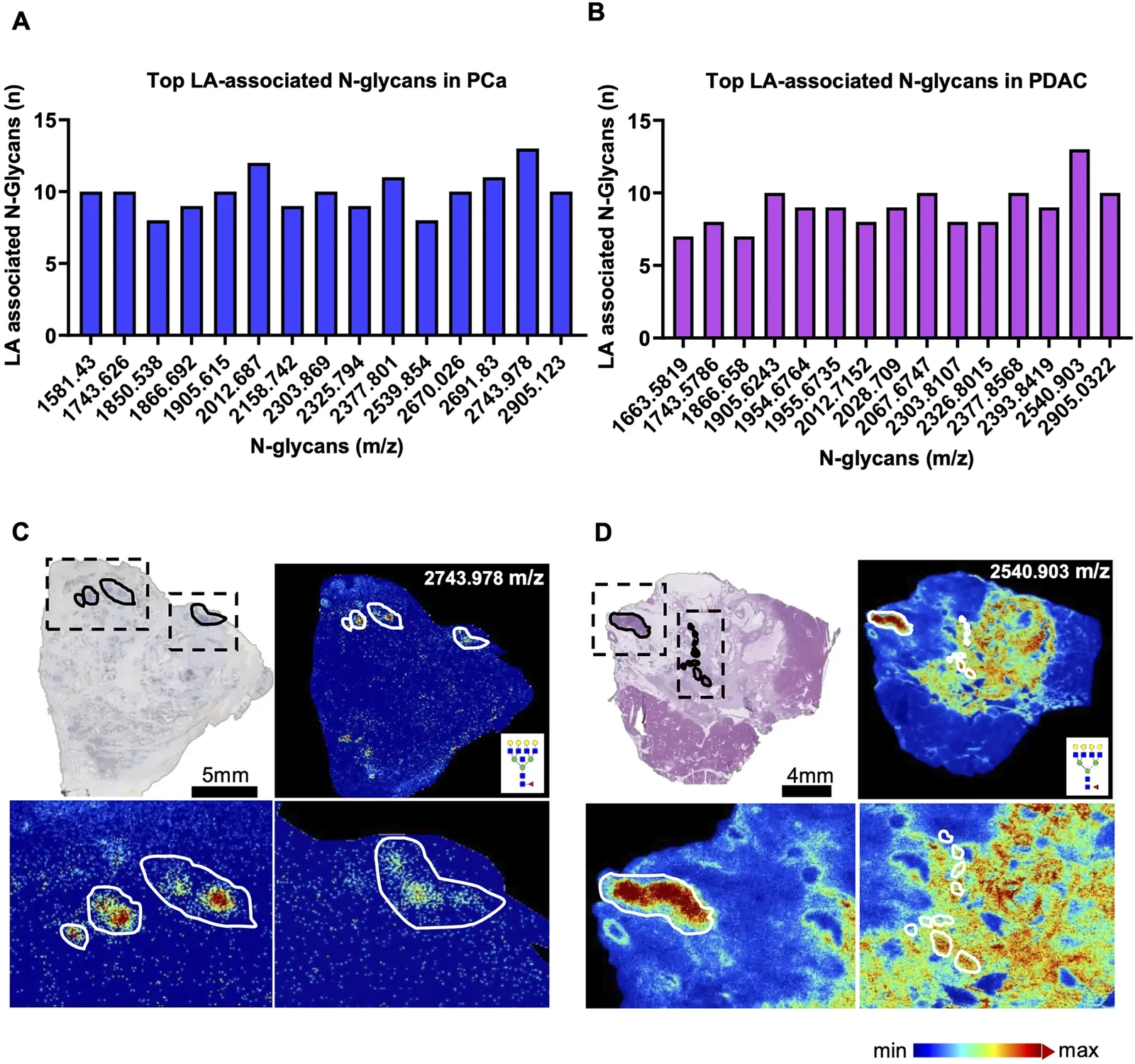
N-glycans co-localized with lymphoid aggregates. The 15 most recurrent N-glycans among the top 25 positive correlates per tissue are shown for **(A)** PCa and **(B)** PDAC. Representative spatial distribution images showing the most frequently detected N-glycan colocalized with LA in **(C)** PCa13 and **(D)** PDAC17 tissues.

The observed differences in N-glycan frequencies within LAs between PCa and PDAC likely reflect differences in the immune cell subtypes comprising these aggregates. To assess immune marker distribution, MALDI-IHC with photocleavable mass tags (42). MALDI-IHC with photocleavable mass tags (42) was performed on available LA-containing tissues from each tumor type (PCa, n = 6; PDAC, n = 8). A panel of mass-tagged antibodies targeting immune cell markers (CD20, CD44, CD3, CD8, CD11b, and CD11c) and other proteins (Collagen 1A1, E-cadherin, vimentin) were used for labeling. UV irradiation was then used to release peptide tags for detection by MALDI MSI. Representative multiplex images are shown in **Figures 3A–C** and **Supplementary Figure 8**, with their corresponding mass spectra and probe panel with *m/z* values are presented in **Figures 3B, C**. Analysis of the immune marker panel showed that signals corresponding to CD20 (*m/z* 997.519), CD3 (*m/z* 1216.735), and CD44 (*m/z* 1102.580) were consistently detected within LAs across both tumor types. Because CD44 is expressed by multiple immune cell populations and a marker of stemness, its signal may represent a heterogeneous mixture of cell types (51). These results were anticipated, as tertiary lymphoid structures identified in other cancers are predominantly composed of both B and T lymphocytes. In a representative tissue sample (**Figures 3D, E**), in PCa, CD20 and CD44 probe signals were predominantly localized to the central regions of LAs, whereas CD3 signal was enriched along the outer margins of the aggregates. CD8 and CD11b signals were detected at lower levels (**Figures 3E, F**). For PDAC (**Figures 3G–I**), CD20, CD44 and CD3 were similarly detected in LAs and in adjacent lymph nodes with CD20 and CD44 more centrally located within each cluster and CD3 signal at the periphery. Additionally, MALDI-IHC staining patterns of LAs followed similar patterns to that of secondary follicles within adjacent lymph nodes (**Figure 3G**; **Supplementary Figure 8A**) and the previously featured lymph node common bile duct metastasis (**Supplementary Figures 8B, C**). Shown in **Supplementary Figures 8D, E**, four more tissue samples, two PCa and two PDAC, contained LAs with staining patterns previously observed including a dominance of CD20, CD44, and CD3 positive staining. Although identical probes were applied across patient samples for each tumor type, the spatial distribution of proteins varied, reflecting inter-patient and intra-tumoral heterogeneity (**Supplementary Figures 8D, E**).

**Figure 3:**
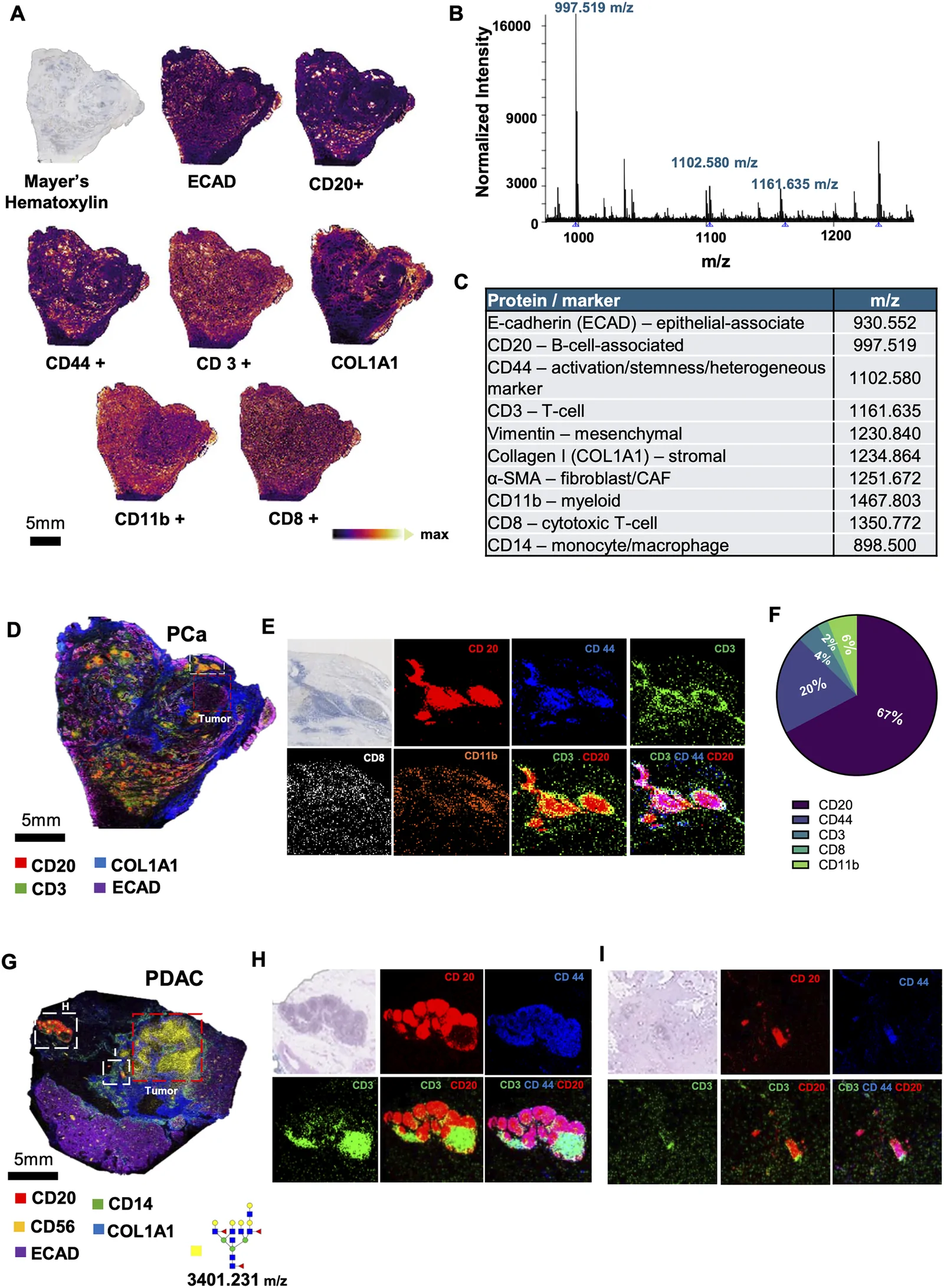
MALDI-IHC spatial profiling of immune cell populations in PCa and PDAC. **(A)** Ion images showing the spatial distribution of immune markers in PCa13. **(B)** Representative mass spectra with annotated protein peaks. **(C)** AmberGen probes used for MALDI-IHC. **(D)** MALDI-IHC image of PCa13 showing CD3 (green), CD20 (red), collagen I (COL1A1; blue), and E-cadherin (purple). **(E)** Mayers hematoxylin and MALDI-IHC image of a prostate cancer LA showing CD20 (red), CD44 (blue), CD3 (green), CD8^+^ (white), and CD11b (orange). **(F)** Quantification of immune cell populations shown in **(E)**. **(G)** MALDI-IHC image of PDAC17 LA showing CD20 (red), CD56 (orange), CD14 (green), E-cadherin (purple), collagen I (COL1A1; blue), and mucin-associated glycan (yellow). **(H, I)** H&E-stained image and corresponding MALDI-IHC of a PDAC-adjacent lymph node and lymphoid aggregate with CD20 (red), CD44 (blue), and CD3 (green).

The relationship between N-glycosylation and immune cell state is bidirectional (52): pro-inflammatory cytokines from immune cells can affect sugar metabolism, glycosidase and glycosyltransferase enzyme expression, which in turn alters the N-glycan biosynthetic pathway (53). Conversely, N-glycans present on TME components and the cancer cells themselves can prevent immune receptor binding or mask cancer cells, preventing immune cells from recognizing them (54–56). Using N-glycans as a correlate to metabolic activity within each LAs, the proximity of LAs to the tumor could also be a factor in whether N-glycans are detected. Characterizing the proximity of LAs to the tumor and TME by spatial omic methods is an ongoing area of research for multiple tumor types (57–63). This was assessed for several PCa tissues which displayed the most distinct LAs and N-glycan expression. Using the Ion Image Mapper feature in the SCiLS software and PDAC13 tissue, N-glycan and immune cell spectral images were co-registered. Four fucosylated N-glycans (m/z = 2743.978, 2539.854, 2905.123, and 2158.742) including tetra-antennary and bi-antennary bisecting species, were found to co-localize with CD20and CD44. For CD44, N-glycans at 2743.836 m/z and 2158.779 m/z, although also detected in stromal regions, were predominantly associated with LAs. In contrast, while present in some LAs, the tetra-antennary N-glycan at m/z = 2539.767 m/z was predominantly restricted to tumor cells, and the m/z= 2905.086 was mainly localized to the stroma. Overall, N-glycan signals demonstrated spatial heterogeneity that correlated with both cell type and proximity to tumor regions. Among these N-glycans, 2743.836 m/z appeared to correspond to most CD20, whereas 2539.797 m/z was associated with both tumor and immune cells. The N-glycan signal was not uniform across all LAs; for example, 2905.086 m/z and 2158.779 m/z were present in LAs shown in region (i) but absent in region(ii) (**Figures 4A, B**). Similar N-glycan distribution was seen in PCa8 (**Supplementary Figures 7A, B**).

**Figure 4:**
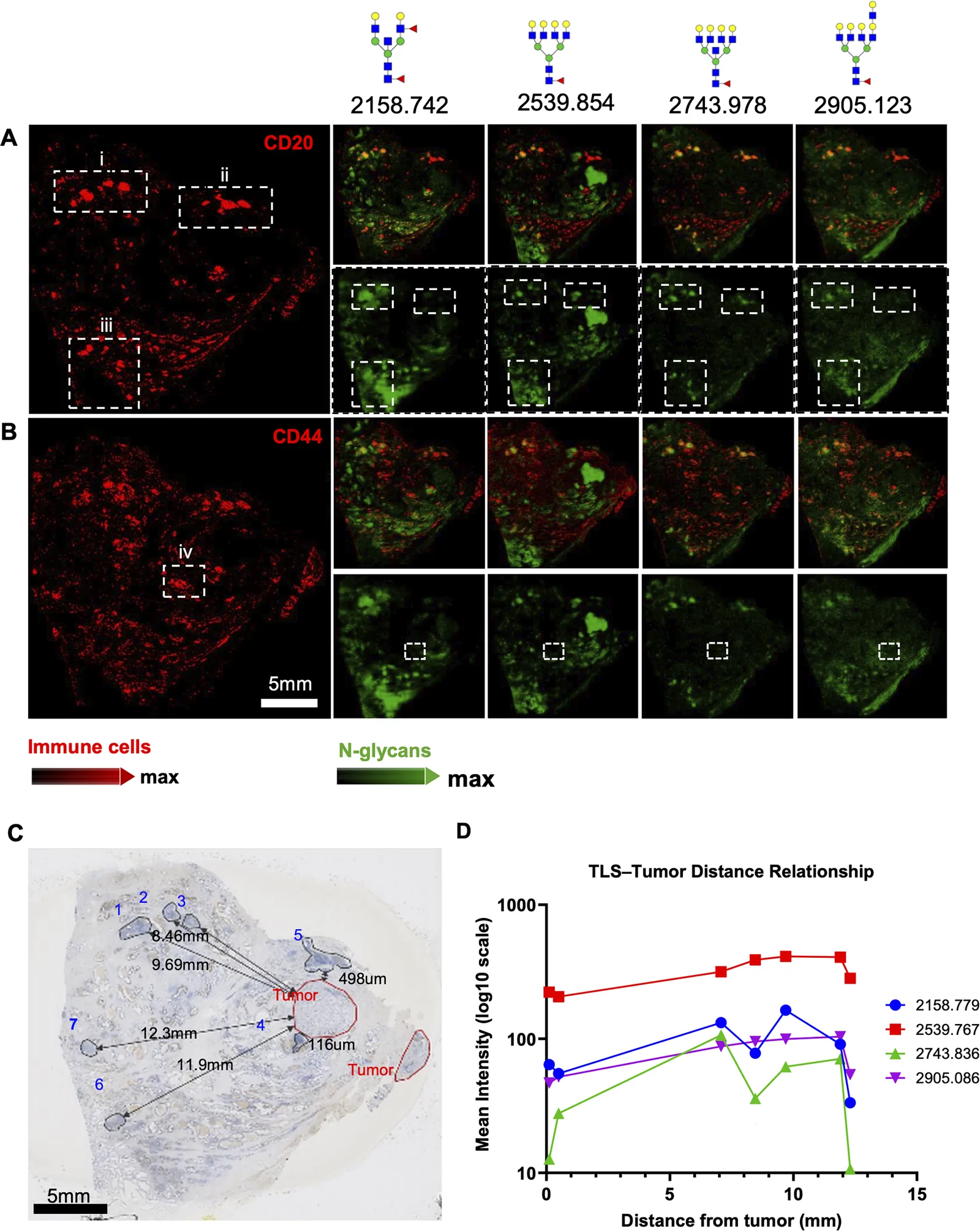
Spatial mapping of N-glycans and immune marker relative to the tumor boundary in one PCa tissue. **(A, B)** Co-registered images of four N-glycans (m/z 2158.742, 2539.854, 2743.978, and 2905.123; green) overlaid with the two predominant immune cell populations, CD20 and CD44 (red), in PCa13 tissue. **(C)** Annotated PCa13 tissue section showing seven lymphoid aggregates (LA1 -LA7) and their proximity to the tumor boundary. **(D)** Mean N-glycan intensity (log_10_ scale; y-axis) plotted against lymphoid aggregate distance from the tumor boundary in millimeters (x-axis).

Further, imaging annotation software was used to measure the LA distances to the nearest tumor boundary (**Figure 4C**). Among the seven lymphoid aggregates evaluated in representative tissue (**Figure 4C**), m/z 2539.767 and 2905.086 exhibited an overall increase in intensity with increasing distance from the tumor, with the highest intensities in lymphoid aggregates located approximately 9–11 mm from the tumor. Reduced intensities were observed in the two most distal lymphoid aggregates (~12 mm). In contrast, m/z 2158.779 and 2743.836 generally increased with distance from the tumor up to approximately 7 mm, after which fluctuations in N-glycan intensity were observed among LAs.(**Figure 4D**). In a second representative tissue section (**Supplementary Figure 9C**), where LAs were located near tumor periphery (0-5.5 mm), less pronounced distance-dependent differences in N-glycan intensity were observed. The mean intensities of m/z 2158.779 and 2743.836 within LAs showed a slight increase with increasing distance from the tumor, whereas m/z 2539.767 fluctuated across lymphoid aggregates and m/z 2905.086 had a slight decrease across distance.

## Discussion

Prior studies, including work from our group, have mapped N-glycans to histopathological features in solid tumors (39, 45, 64–66). However, no study to date has characterized the spatial relationship between the MSI detected N-glycans within and surrounding lymphoid aggregates (LAs) in PCa and PDAC tissues. Addressing this gap may provide insight into the spatial glycomic heterogeneity of LAs in these cancers, which remain largely refractory to immunotherapy (67, 68). Although LAs have been associated with improved prognosis in several tumor types, including colorectal (27, 28), lung (29), and breast (20) cancers, not all patients with LAs will achieve improved prognosis (69, 70) or respond to immunotherapy. This variability may arise from LA heterogeneity (71), differences in the surrounding extracellular matrix in different tissue types, and variation in glycosylation-related metabolic activity (68, 72). In this study, multimodal MALDI-MSI and MALDI-IHC platform enabled spatial profiling of LA features, revealing distinct N-glycan signatures and immune architectures. This multimodal approach revealed spatial heterogeneity in LA-associated N-glycan signatures and provides a foundation for future studies evaluating whether these N-glycan patterns relate to immune activity or functional states within LAs.

When detected in LAs, N-glycans represented three structural groups: oligo-mannose compositions, fucosylated tetra-antennary with or without N-acetylglucosamine additions, and bisecting N-acetylglucosamine in fucosylated bi-, tri- and tetra-antennary compositions (**Supplementary Table 3**). These structural groups are consistent with known N-glycan biosynthetic pathways. For example, bisecting GlcNAc-containing structures are generated through MGAT3-mediated GlcNAc addition, whereas increased branching is associated with enzymes such as MGAT5. In contrast, oligo-mannose structures are shaped by mannose-processing enzymes, including GANAB and MAN1A1, during high-mannose glycan maturation (73). The Pearson correlation and frequency analyses further supported the presence of recurrent LA-associated N-glycan patterns across both tumor types. Although the overall correlation values were modest, most structural categories showed positive tissue-level median correlations with LA regions, indicating that multiple N-glycan classes contributed to LA-associated signal rather than one dominant glycan type. Identifying LAs with detectable N-glycan signal provides a basis for future studies comparing these regions with LAs that show limited or absent N-glycan signal. Spatial transcriptomic studies for PCa (57, 58, 61, 62) and PDAC (59, 60, 63, 74) have characterized tumor–immune organization and immune-related gene expression patterns within the tumor microenvironment, highlighting the importance of spatially resolved approaches for defining immune heterogeneity in these tumor types. In this context, N-glycan MALDI-MSI may provide a complementary framework for evaluating LA-associated N-glycan heterogeneity and guiding future single-cell, proteomic, and spatial multi-omic analyses.

Previous studies provide mechanistic evidence that N-glycan structures can influence immune cell development and function. Oligo-mannose-type N-glycans have been associated with ER glycoprotein processing and mannose-dependent recognition by innate immune lectin receptors, linking altered N-glycan processing to immune recognition pathways (75, 76). Also, a decrease in bisecting GlcNAc structures have been linked to the polarization of resting macrophages M0 into pro-inflammatory M1 (77), while an increase in bisecting GlcNAc promotes M2 anti-inflammatory polarization (78). Another study targeting Mgat1, the gene responsible for the biosynthetic switch of high-mannose to complex N-glycans, demonstrated that the development and survival of pre-B and immature B cells are impaired in Mgat1-deficient mice, highlighting the importance of GlcNAc branching in B cell development (79). In T cells, overexpression of highly branched glycans and fucosylation strengthens PD-1/PD-L1 binding, reducing the efficacy of anti-PD-L1 immunotherapy in ovarian tumors (80). Similarly, in a gastrointestinal tumor study, complex branched N-glycans promote an immunosuppressive phenotype by inhibiting IFNγ production (81). Together, these studies show that glycosylation can influence immune cell biology. While the N-glycan patterns identified in this study may reflect underlying biological differences among lymphoid aggregates, the current data establish spatial associations and do not define the functional significance of these glycan signatures.

The sizes of observed LAs in both tumor types exhibited measurements comparable to those measured in lymph nodes and to previously reported gastric (82) and colorectal (27) tumors. LAs exhibiting N-glycan signal were characterized by central CD20 and CD44 signals, surrounded by peripheral CD3 signal, with lower levels of CD8 and CD11b signals detected throughout the aggregates, consistent with the architecture of primary follicle-like LA (22, 83). While the same immune cell types and ECM components were detected across both tumor types, their N-glycan expression profiles showed two distinct levels of variability: spatial heterogeneity within individual tissues and differences between patient samples. Within a single PCa tissue section, lymphoid aggregates located at different distances from the tumor exhibited variable N-glycan intensities. Although aggregates farther from the tumor generally showed higher N-glycan abundance, two of the most distal aggregates displayed lower intensities despite their greater distance. These findings highlight spatial heterogeneity in N-glycan expression among lymphoid aggregates but should be considered exploratory observations from a single tissue specimen. Additional studies are needed to determine whether these patterns are consistently associated with tumor proximity or other local microenvironmental factors. Tumor cells generally require a vast amount of glucose to sustain growth and survival, which strains glycolysis and other metabolic pathways. Because N-glycan biosynthesis depends on glucose and glutamine to produce nucleotide sugar donors that supply activated monosaccharides for stepwise enzymatic elongation by families of glycosyltransferases, metabolic changes can influence the structure and abundance of N-glycans. Such alterations have the potential to influence N-glycan biosynthesis and glycan composition within both tumor and immune cell populations ( (30). Among the key metabolites, UDP-galactose and UDP-N-acetylglucosamine, derived from the Leloir pathway and the hexosamine biosynthetic pathway, respectively, provide the galactose and N-acetylglucosamine (GlcNAc) residues required for glycan assembly and maturation (84–86). As such, it can be presumed that N-glycan enrichment or depletion in LA and neighboring ECM may reflect early metabolic changes associated with immune cell suppression or activation. These observations suggest that LA-associated glycan patterns may reflect local microenvironmental variation, but functional studies will be required to determine their biological significance. Based on these observations, we propose a working model in which immune-cell recruitment and LA formation in cold tumors are accompanied by spatially variable N-glycan patterns within and around LAs (**Figure 5**). These patterns may reflect differences in local glycan biosynthesis, immune-cell composition and tumor proximity. These observations support a working model in which LA formation in cold tumors is accompanied by spatially variable N-glycan remodeling within and around immune aggregates (**Figure 5**). This model provides a framework for future studies testing whether LA-associated glycan patterns reflect differences in immune-cell composition, local metabolism, or functional immune state.

**Figure 5:**
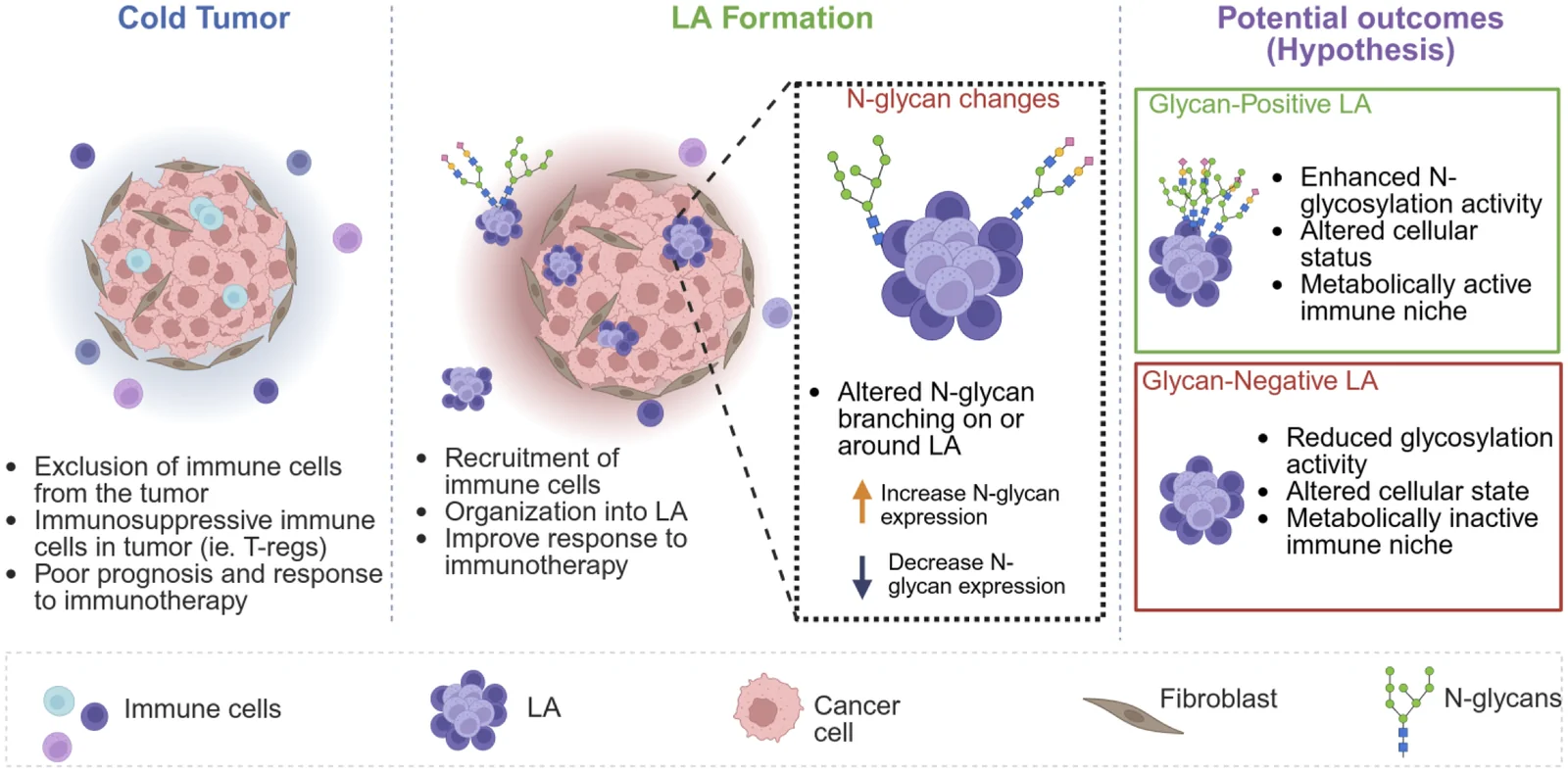
Hypothesized relationship between lymphoid aggregate formation and N-glycan remodeling in cold tumors. Cold tumors are characterized by immune exclusion and immunosuppressive microenvironments. N-glycan patterns within and surrounding LAs may reflect local differences in glycan biosynthesis, immune-cell composition, and microenvironmental context. We propose that differences in LA-associated N-glycan detectability may indicate distinct immune microenvironments, but further studies are needed to determine their relationship to immune function and metabolic state.

Overall, this study was designed to capture intra-patient spatial glycomic variation, representing a strength through high-resolution analysis of LA heterogeneity and the integration of N-glycan MSI with MALDI-IHC to map glycan composition alongside immune architecture. However, several limitations should be considered. Only a subset of screened tissues met inclusion criteria and were analyzed for LA-associated N-glycan patterns (PCa, n = 14 of 49; PDAC, n = 17 of 26), potentially biasing the analysis toward higher-abundance glycans and not fully capturing LA diversity. This limited sample set also restricts comparative analyses between prostate and pancreatic cancers, particularly in identifying disease-specific differences. In addition, MALDI-IHC was also performed on available LA-containing tissues only (PCa, n = 6; PDAC, n = 8), limiting the extent to which immune marker patterns could be compared across the full N-glycan-analyzed set. Spatial associations between N-glycans, immune markers, and tumor proximity are correlative and do not establish functional mechanisms. The absence of matched TLS-positive and TLS-negative comparisons further limits the ability to define TLS-specific glycomic features independent of interpatient variability, and the lack of treatment response data precludes assessment of predictive relevance. Overall, these findings are based on LAs with detectable N-glycan signals and should therefore be interpreted as exploratory pending validation in larger, clinically annotated cohorts with functional validation.

Together our findings demonstrate spatial heterogeneity in LA-associated N-glycan profiles within PCa and PDAC tissues. The abundance, composition, and spatial distribution of N-glycans varied among LAs and surrounding tissues regions, highlighting differences in glycan localization across the tumor microenvironment. These results demonstrate the feasibility of spatial glycomic profiling of LAs and identify candidate glycan patterns. As such, they should be considered exploratory and hypothesis-generating and provide a framework for future studies integrating spatial glycomics with cellular and clinical data to evaluate the biological and potential clinical relevance of LA-associated N-glycan patterns.

## Data Availability

The raw data supporting the conclusions of this article will be made available by the authors, without undue reservation.

## Supplementary material

Supplementary Table 1 Clinicopathologic characteristics of the prostate cancer patient cohort, including sample ID, body mass index (BMI), prostate-specific antigen (PSA) level, Gleason score, and pathologic stage.

Supplementary Table 2 Clinicopathologic characteristics of the pancreatic cancer patient cohort, including tissue block number (TB#), race, sex, age, recurrence status, type of recurrence, histological type, histological grade, and tumor stage.

Supplementary Table 3 Lymphoid Aggregate N-glycan Peak list. Listed are the detected N-glycan masses (m/z), composition and structures linked to the Pearson correlation coefficient calculations and N-glycan frequency comparisons as highlighted in **Figures 2A, B**.

Supplementary Figure 1 Tissue screening and inclusion workflow for LA-associated N-glycan analysis. PCa (n = 49) and PDAC (n = 26) tissues were screened for histologically identifiable lymphoid aggregates >40 µm in size in width. Tissues with absent, dispersed, poorly defined, or undersized LAs, or insufficient LA-associated N-glycan signal, were excluded. PCa (n = 14) and PDAC (n = 17) tissues met inclusion criteria and were analyzed downstream.

Supplementary Figure 2 Size and distribution of lymphoid aggregates across tumor types and a positive control lymph node. Mayer’s hematoxylin staining of LA in **(A)** prostate cancer (PCa6) and **(B)** pancreatic ductal adenocarcinoma (PDAC15) tissues. Red outlines indicate representative LA measurements and their distribution relative to the tumor regions. PDAC lymphoid aggregates display features consistent with germinal center-like organization, with a lightly stained central region surrounded by a darker peripheral zone. **(C)** Positive control lymph node from PDAC8 showing pale germinal centers encircled by a darker mantle zone. Scale bars = 100 µm–5 mm.

Supplementary Figure 3 Examples of PCa and PDAC tissues lacking co-localized N-glycan signal in lymphoid aggregates (LAs). Representative prostate cancer (PCa, top) and pancreatic ductal adenocarcinoma (PDAC, bottom) tissues in which no N-glycan signal co-localized with the LAs. For each tissue, histology of LAs are shown alongside ion images for representative N-glycans at m/z 1581.533, 1743.579, 2377.857, 2539.912, and 2905.032. PDAC has a MALDI-IHC showing immune markers. Scale bars, 3 mm.

Supplementary Figure 4 Pearson correlation analysis of N-glycans within lymphoid aggregates in prostate cancer and pancreatic ductal adenocarcinoma. **(A–N)** Pearson correlation analyses of the top 25 N-glycans detected within LAs in PCa. **(O–AE)** Pearson correlation analyses of the top 25 N-glycans detected within LA in PDAC.

Supplementary Figure 5 N-glycan-based segmentation identifies lymphoid aggregates in prostate cancer and pancreatic ductal adenocarcinoma tissues. N-glycan-based tissue segmentation of **(A)** PCa13 and **(B)** PDAC17. **(C, D)** Hierarchical *k*-means clustering displayed as Manhattan plots, illustrating relationships among segmented tissue regions. Unsupervised segmentation based on the distributions of 78 detected N-glycans identified distinct spectral clusters corresponding to lymphoid aggregates, comprising 8,265 pixels in prostate cancer (yellow) and 10,174 pixels in pancreatic ductal adenocarcinoma and the adjacent lymph node (orange).

Supplementary Figure 6 Representative N-glycan MSI of a prostate cancer and lymphoid aggregate (PCa6). **(A)** Mayer’s hematoxylin-stained section showing the lymphoid aggregate (LA; blue outline) and adjacent tumor region (red outline). **(B)** Control ion image showing N-glycans absent from the LA. Representative stromal N-glycans are highlighted in aqua (m/z 2289; Hex5HexNAc4NeuAc2) and blue (m/z 2122; Hex5HexNAc4Fuc1NeuAc1), whereas representative tumor-associated N-glycans are highlighted in red (m/z 2685; Hex7HexNAc6Fuc2 and m/z 2978; Hex7HexNAc6Fuc4). **(C–I)** Representative LA-associated N-glycans: **(C)** m/z 2743 (Hex7HexNAc7Fuc1), **(D)** m/z 2377 (Hex5HexNAc6Fuc1), **(E)** m/z 2905 (Hex8HexNAc7Fuc1), **(F)** m/z 2012 (Hex5HexNAc5Fuc1), **(G)** m/z 1905 (Hex9HexNAc2), **(H)** m/z 3271 (Hex9HexNAc8Fuc1), and **(I)** m/z 2539 (Hex7HexNAc6Fuc1).

Supplementary Figure 7 Representative N-glycan MSI of a pancreatic ductal adenocarcinoma and lymphoid aggregate (PDAC16). **(A)** Mayer’s hematoxylin-stained section showing the lymphoid aggregate (LA; blue outline) and the adjacent tumor region (red outline, inset). **(B)** Control ion image showing N-glycans absent from the LA. A representative stromal N-glycan is highlighted in blue (m/z 1976; Hex5HexNAc4NeuAc1), whereas a representative tumor-associated N-glycan is highlighted in red (m/z 3109; Hex&HexNAc8Fuc1). **(C–H)** Representative LA-associated N-glycan ion images: **(C)** m/z 2377 (Hex5HexNAc6Fuc1), **(D)** m/z 2539 (Hex7HexNAc6Fuc1), **(E)** m/z 1905 (Hex9HexNAc2), **(F)** m/z 2743 (Hex7HexNAc7Fuc1), **(G)** m/z 2012 (Hex5HexNAc5Fuc1), and **(H)** m/z 2905 (Hex8HexNAc7Fuc1).

Supplementary Figure 8 MALDI-IHC spatial profiling of lymphoid aggregates and lymph nodes in pancreatic ductal adenocarcinoma and prostate cancer. **(A)** Representative ion images showing the spatial distribution of immune cell markers and extracellular matrix (ECM) proteins in PDAC17 tissue. **(B)** Mayer’s hematoxylin-stained section of a PDAC-associated lymph node demonstrating organized lymphoid architecture. **(C)** Corresponding MALDI-IHC image of the lymph node showing the spatial distribution of immune cell markers. **(D)** Representative MALDI-IHC images of additional prostate cancer tissues, PCa1 (left) and PCa6 (right), demonstrating similar immune cell and stromal marker distributions within lymphoid aggregates. **(E)** Representative MALDI-IHC images of additional pancreatic ductal adenocarcinoma tissues, PDAC16 (left) and PDAC2 (right), demonstrating similar immune cell and stromal marker distributions within lymphoid aggregates, including COL1A1, CD3/CD8, and CD20^+^.

Supplementary Figure 9 Spatial mapping of N-glycans and immune marker relative to the tumor boundary in one PCa tissue (PCa8). **(A, B)** Co-registered images of four N-glycans (m/z 2158.742, 2539.854, 2743.978, and 2905.123; green) overlaid with the two predominant immune cell populations, CD20 and CD44 (red), in PCa8 tissue. **(C)** Annotated PCa8 tissue section showing the locations of three lymphoid aggregates (LA1–LA3) relative to the tumor boundary. **(D)** Relationship between lymphoid aggregate distance from the tumor boundary (y-axis, mm) and mean N-glycan intensity (x-axis).

## References

[1] Rawla P . Epidemiology of prostate cancer. World J Oncol. (2019) 10:63–89. doi: 10.14740/wjon1191 31068988 PMC6497009

[2] Kanno A Masamune A Hanada K Kikuyama M Kitano M . Advances in early detection of pancreatic cancer. Diagnostics (Basel). (2019) 9:18. doi: 10.3390/diagnostics9010018 30764550 PMC6468351

[3] Venkatachalam S McFarland TR Agarwal N Swami U . Immune checkpoint inhibitors in prostate cancer. Cancers (Basel). (2021) 13:2187. doi: 10.3390/cancers13092187 34063238 PMC8125096

[4] Silva LGO Lemos FFB Luz MS Rocha Pinheiro SL Calmon MDS Correa Santos GL . New avenues for the treatment of immunotherapy-resistant pancreatic cancer. World J Gastrointest Oncol. (2024) 16:1134–53. doi: 10.4251/wjgo.v16.i4.1134 38660642 PMC11037047

[5] Ju Y Xu D Liao M Sun Y Bao W Yao F . Barriers and opportunities in pancreatic cancer immunotherapy. NPJ Precis Oncol. (2024) 8:199. doi: 10.1038/s41698-024-00681-z 39266715 PMC11393360

[6] Šamija I Fröbe A . Challenges in manipulating immune system to treat prostate cancer. Acta Clin Croat. (2019) 58:76–81. doi: 10.20471/acc.2019.58.s2.13 PMC8693557 34975203

[7] Topalian SL Hodi FS Brahmer JR Gettinger SN Smith DC McDermott DF . Safety, activity, and immune correlates of anti-PD-1 antibody in cancer. N Engl J Med. (2012) 366:2443–54. doi: 10.1056/nejmoa1200690 22658127 PMC3544539

[8] Sridaran D Bradshaw E DeSelm C Pachynski R Mahajan K Mahajan NP . Prostate cancer immunotherapy: Improving clinical outcomes with a multi-pronged approach. Cell Rep Med. (2023) 4:101199. doi: 10.1016/j.xcrm.2023.101199 37738978 PMC10591038

[9] Tsumura A Levis D Tuscano JM . Checkpoint inhibition in hematologic malignancies. Front Oncol. (2023) 13:2023. doi: 10.3389/fonc.2023.1288172 37920162 PMC10619902

[10] Carlino MS Larkin J Long GV . Immune checkpoint inhibitors in melanoma. Lancet. (2021) 398:1002–14. doi: 10.1016/s0140-6736(21)01206-x 34509219

[11] Tang S Qin C Hu H Liu T He Y Guo H . Immune checkpoint inhibitors in non-small cell lung cancer: Progress, challenges, and prospects. Cells. (2022) 11:320. doi: 10.3390/cells11030320 35159131 PMC8834198

[12] Kim H Choi JM Lee KM . Immune checkpoint blockades in triple-negative breast cancer: Current state and molecular mechanisms of resistance. Biomedicines. (2022) 10:1130. doi: 10.3390/biomedicines10051130 35625867 PMC9138553

[13] Lasorsa F di Meo NA Rutigliano M Milella M Ferro M Pandolfo SD . Immune checkpoint inhibitors in renal cell carcinoma: Molecular basis and rationale for their use in clinical practice. Biomedicines. (2023) 11:1071. doi: 10.3390/biomedicines11041071 37189689 PMC10136190

[14] Damodaran S Kyriakopoulos CE Jarrard DF . Newly diagnosed metastatic prostate cancer: Has the paradigm changed? Urol Clin North Am. (2017) 44:611–21. doi: 10.1016/j.ucl.2017.07.008 29107277 PMC6402766

[15] Bonaventura P Shekarian T Alcazer V Valladeau-Guilemond J Valsesia-Wittmann S Amigorena S . Cold tumors: A therapeutic challenge for immunotherapy. Front Immunol. (2019) 10:2019. doi: 10.3389/fimmu.2019.00168 30800125 PMC6376112

[16] Ouyang P Wang L Wu J Tian Y Chen C Li D . Overcoming cold tumors: a combination strategy of immune checkpoint inhibitors. Front Immunol. (2024) 15:1344272. doi: 10.3389/fimmu.2024.1344272 38545114 PMC10965539

[17] Galon J Bruni D . Approaches to treat immune hot, altered and cold tumours with combination immunotherapies. Nat Rev Drug Discov. (2019) 18:197–218. doi: 10.1038/s41573-018-0007-y 30610226

[18] Minogue E Baldominos P Hsu L Haigis MC Agudo J . Decoding the spatial dynamics of tumor and immune cell interactions in solid cancers. Cancer Cell. (2026) 44:1–5. doi: 10.1016/j.ccell.2025.10.007 41202809

[19] Lehmann J Thelen M Kreer C Schran S Garcia-Marquez MA Cisic I . Tertiary lymphoid structures in pancreatic cancer are structurally homologous, share gene expression patterns and B-cell clones with secondary lymphoid organs, but show increased T-cell activation. Cancer Immunol Res. (2025) 13:323–36. doi: 10.1158/2326-6066.cir-24-0299 39661055

[20] Wang L Gong S Pang L Zhang S Zhang X He W . Genomic properties and clinical outcomes associated with tertiary lymphoid structures in patients with breast cancer. Sci Rep. (2023) 13:13542. doi: 10.1038/s41598-023-40042-7 37598257 PMC10439954

[21] Zhao L Jin S Wang S Zhang Z Wang X Chen Z . Tertiary lymphoid structures in diseases: immune mechanisms and therapeutic advances. Signal Transduction Targeted Ther. (2024) 9:225. doi: 10.1038/s41392-024-01947-5 39198425 PMC11358547

[22] Sato Y Silina K van den Broek M Hirahara K Yanagita M . The roles of tertiary lymphoid structures in chronic diseases. Nat Rev Nephrol. (2023) 19:525–37. doi: 10.1038/s41581-023-00706-z 37046081 PMC10092939

[23] Zhao R Zhang J Ma J Qu Y Yang Z Yin Z . cGAS-activated endothelial cell–T cell cross-talk initiates tertiary lymphoid structure formation. Sci Immunol. (2024) 9:eadk2612. doi: 10.1126/sciimmunol.adk2612 39093956

[24] Liu X Lv W Huang D Cui H . The predictive role of tertiary lymphoid structures in the prognosis and response to immunotherapy of lung cancer patients: a systematic review and meta-analysis. BMC Cancer. (2025) 25:87. doi: 10.1186/s12885-025-13484-7 39815237 PMC11734324

[25] Trüb M Zippelius A . Tertiary lymphoid structures as a predictive biomarker of response to cancer immunotherapies. Front Immunol. (2021) 12:674565. doi: 10.3389/fimmu.2021.674565 PMC8149953 34054861

[26] Williams CG Lee HJ Asatsuma T Vento-Tormo R Haque A . An introduction to spatial transcriptomics for biomedical research. Genome Med. (2022) 14:68. doi: 10.1186/s13073-022-01075-1 35761361 PMC9238181

[27] Schweiger T Berghoff AS Glogner C Glueck O Rajky O Traxler D . Tumor-infiltrating lymphocyte subsets and tertiary lymphoid structures in pulmonary metastases from colorectal cancer. Clin Exp Metastasis. (2016) 33:727–39. doi: 10.1007/s10585-016-9813-y 27449756 PMC5035322

[28] Di Caro G Bergomas F Grizzi F Doni A Bianchi P Malesci A . Occurrence of tertiary lymphoid tissue is associated with T-cell infiltration and predicts better prognosis in early-stage colorectal cancers. Clin Cancer Res. (2014) 20:2147–58. doi: 10.1158/1078-0432.ccr-13-2590 24523438

[29] Weng Y Yuan J Cui X Wang J Chen H Xu L . The impact of tertiary lymphoid structures on tumor prognosis and the immune microenvironment in non-small cell lung cancer. Sci Rep. (2024) 14:16246. doi: 10.1038/s41598-024-64980-y 39009684 PMC11250816

[30] Pinho SS Macauley MS Läubli H . Tumor glyco-immunology, glyco-immune checkpoints and immunotherapy. J Immunother Cancer. (2025) 13:e012391. doi: 10.1136/jitc-2025-012391 40533266 PMC12182193

[31] Möckl L . The emerging role of the mammalian glycocalyx in functional membrane organization and immune system regulation. Front Cell Dev Biol. (2020) 8:253. doi: 10.3389/fcell.2020.00253 PMC7174505 32351961

[32] Pasala C Sharma S Roychowdhury T Moroni E Colombo G Chiosis G . N-glycosylation as a modulator of protein conformation and assembly in disease. Biomolecules. (2024) 14:282. doi: 10.3390/biom14030282 38540703 PMC10968129

[33] Rabinovich GA Rademacher C Schattner M Macauley MS . Glycan-binding proteins in immunity. Annu Rev Immunol. (2026) 44(1):267–94. doi: 10.1146/annurev-immunol-083024-030822 41729700

[34] Läubli H Varki A . Sialic acid-binding immunoglobulin-like lectins (Siglecs) detect self-associated molecular patterns to regulate immune responses. Cell Mol Life Sci. (2020) 77:593–605. doi: 10.1007/s00018-019-03288-x PMC7942692 31485715

[35] Mantuano NR Läubli H . Sialic acid and Siglec receptors in tumor immunity and immunotherapy. Semin Immunol. (2024) 74-75:101893. doi: 10.1016/j.smim.2024.101893 39427573

[36] Liu FT Stowell SR . The role of galectins in immunity and infection. Nat Rev Immunol. (2023) 23:479–94. doi: 10.1038/s41577-022-00829-7 36646848 PMC9842223

[37] Hartig JP Bejar K Young LEA Grimsley G Bethard JR Troyer DA . Determining the N-glycan and collagen/extracellular matrix protein compositions in a novel outcome cohort of prostate cancer tissue microarrays using MALDI-MSI. Cancer Res Commun. (2024) 4:3036–48. doi: 10.1158/2767-9764.crc-24-0152 39347566 PMC11600299

[38] Ippolito JE Hartig JP Bejar K Nakhoul H Sehn JK Weimholt C . N-linked fucosylated glycans are biomarkers for prostate cancer with a neuroendocrine and metastatic phenotype. Mol Cancer Res. (2025) 23:59–70. doi: 10.1158/1541-7786.mcr-24-0660 39417716 PMC11694069

[39] McDowell CT Klamer Z Hall J West CA Wisniewski L Powers TW . Imaging mass spectrometry and lectin analysis of N-linked glycans in carbohydrate antigen–defined pancreatic cancer tissues. Mol Cell Proteomics. (2021) 20:100012. doi: 10.1074/mcp.ra120.002256 33581409 PMC8724603

[40] Wallace EN West CA McDowell CT Lu X Bruner E Mehta AS . An N-glycome tissue atlas of 15 human normal and cancer tissue types determined by MALDI-imaging mass spectrometry. Sci Rep. (2024) 14:489. doi: 10.1038/s41598-023-50957-w 38177192 PMC10766640

[41] Dunne J Griner J Romeo M Macdonald J Krieg C Lim M . Evaluation of antibody-based single cell type imaging techniques coupled to multiplexed imaging of N-glycans and collagen peptides by matrix-assisted laser desorption/ionization mass spectrometry imaging. Anal Bioanal Chem. (2023) 415:7011–24. doi: 10.1007/s00216-023-04983-2 37843548 PMC10632234

[42] Yagnik G Liu Z Rothschild KJ Lim MJ . Highly multiplexed immunohistochemical MALDI-MS imaging of biomarkers in tissues. J Am Soc Mass Spectrom. (2021) 32:977–88. doi: 10.1021/jasms.0c00473 33631930 PMC8033562

[43] Piyadasa H Oberlton B Ribi M Leow K Ranek JS Averbukh I . Multi-omic landscape of human gliomas from diagnosis to treatment and recurrence. Cancer Cell. (2026) 44:112–28.e6. doi: 10.1016/j.ccell.2025.11.006 41386224 PMC13178252

[44] Jones EE Drake RR Dressman JW Parihar V Stubler R Masters E . Applying imaging mass spectrometry to define the N-glycan profiles of co-localized virus and immune cell infiltrates in post-COVID-19 infected lung autopsy tissues. Front Anal Sci. (2022) 2:2022. doi: 10.3389/frans.2022.1021008

[45] Young LEA Nietert PJ Stubler R Kittrell CG Grimsley G Lewin DN . Utilizing multimodal mass spectrometry imaging for profiling immune cell composition and N-glycosylation across colorectal carcinoma disease progression. Front Pharmacol. (2023) 14:1337319. doi: 10.3389/fphar.2023.1337319 38273829 PMC10808565

[46] Kittrell C Young LEA McDowell C Grimsley G Mehta AS Angel PM . Spatial atlasing of N-glycosylation in healthy control and clear cell renal cell carcinoma tissues linked with immune checkpoint inhibition response by MALDI mass spectrometry imaging. Anal Chim Acta. (2025) 1380:344724. doi: 10.1016/j.aca.2025.344724 41241472 PMC13155251

[47] Dressman JW Bayram MF Angel PM Drake RR Mehta AS . Single-cell multiomic MALDI-MSI analysis of lipids and N-glycans through affinity array capture. Anal Chem. (2025) 97:12493–502. doi: 10.1021/acs.analchem.4c06233 40500282

[48] Drake RR Powers TW Norris-Caneda K Mehta AS Angel PM . In situ imaging of N-glycans by MALDI imaging mass spectrometry of fresh or formalin-fixed paraffin-embedded tissue. Curr Protoc Protein Sci. (2018) 94:e68. doi: 10.17504/protocols.io.36wgqj2j5vk5/v2 30074304

[49] McDowell CT Lu X Mehta AS Angel PM Drake RR . Applications and continued evolution of glycan imaging mass spectrometry. Mass Spectrom Rev. (2023) 42:674–705. doi: 10.1002/mas.21725 34392557 PMC8946722

[50] Lu X McDowell CT Blaschke CRK Liu L Grimsley G Wisniewski L . Bioorthogonal chemical labeling probes targeting sialic acid isomers for N-glycan MALDI imaging mass spectrometry of tissues, cells, and biofluids. Anal Chem. (2023) 95:7475–86. doi: 10.1021/acs.analchem.2c04882 37126482 PMC10193362

[51] Johnson P Ruffell B . Cd44 and its role in inflammation and inflammatory diseases. Inflammation Allergy Drug Targets. (2009) 8:208–20. doi: 10.2174/187152809788680994 19601881

[52] Sun R Kim A Lim S . Glycosylation of immune receptors in cancer. Cells. (2021) 10:1100. doi: 10.3390/cells10051100 34064396 PMC8147841

[53] Radovani B Gudelj I . N-glycosylation and inflammation; the not-so-sweet relation. Front Immunol. (2022) 13:893365. doi: 10.3389/fimmu.2022.893365 35833138 PMC9272703

[54] De Bousser E Meuris L Callewaert N Festjens N . Human t cell glycosylation and implications on immune therapy for cancer. Hum Vaccin Immunother. (2020) 16:2374–88. doi: 10.1080/21645515.2020.1730658 32186959 PMC7644206

[55] Zhu Q Chen X Duan X Sun J Yi W . Targeting glycosylation to enhance tumor immunotherapy. Trends Pharmacol Sci. (2025) 46:863–76. doi: 10.1016/j.tips.2025.07.013 40816979

[56] Rodriguez E . Tumor glycosylation: a main player in the modulation of immune responses. Eur J Immunol. (2025) 55:e202451318. doi: 10.1002/eji.202451318 40071681 PMC11898543

[57] Huang S Ou Y Zhuang W Huang J Wang B Li Z . Mapping the immune landscape of PCa: from tumor microenvironment to therapeutics. Biochim Biophys Acta (BBA) - Rev Cancer. (2026) 1881:189586. doi: 10.1016/j.bbcan.2026.189586 41956336

[58] Xue J Liao K Zhang M . Tumor-immune spatiotemporal co-evolution as a paradigm for overcoming therapy resistance in advanced prostate cancer. Front Immunol. (2026) 17. doi: 10.3389/fimmu.2026.1797299 41890710 PMC13013517

[59] Ohara Y Liu H Moreno P Suzuki S Hussain SP . Molecular, metabolic, and histological subtypes of pancreatic ductal adenocarcinoma and its tumor microenvironment: insights into tumor heterogeneity and clinical implications. Pharmacol Ther. (2026) 277:108946. doi: 10.1016/j.pharmthera.2025.108946 41183744

[60] Bärthel S Falcomatà C Rad R Theis F Saur D . Single-cell profiling to explore pancreatic cancer heterogeneity, plasticity and response to therapy. Nat Cancer. (2023) 4:454–67. doi: 10.1038/s43018-023-00526-x PMC7615362 36959420

[61] Ak Ç Sayar Z Thibault G Burlingame E Kuykendall M Eng J . Multiplex imaging of localized prostate tumors reveals altered spatial organization of AR-positive cells in the microenvironment. iScience. (2024) 27:110668. doi: 10.1016/j.isci.2024.110668 39246442 PMC11379676

[62] Calagua C Ficial M Jansen C Hirz T Del Balzo L Wilkinson S . A subset of localized prostate cancer displays an immunogenic phenotype associated with losses of key tumor suppressor genes. Clin Cancer Res. (2021) 27:4836–47. doi: 10.1158/1078-0432.ccr-21-0121 34168052 PMC8416924

[63] Yousuf S Qiu M Voith von Voithenberg L Hulkkonen J Macinkovic I Schulz A . Spatially resolved multi-omics single-cell analyses inform mechanisms of immune dysfunction in pancreatic cancer. Gastroenterology. (2023) 165:891–908.e14. doi: 10.1053/j.gastro.2023.05.036 37263303

[64] Hartig J Young L Grimsley G Mehta A Ippolito J Leach R . The glycosylation landscape of prostate cancer tissues and biofluids. In: Advances in Cancer Research. vol 161. Cambridge MA: Academic Press. (2024). pp 1–30. 10.1016/bs.acr.2024.04.005 39032948

[65] Blaschke C McDowell C Black A Mehta A Angel P Drake R . Glycan imaging mass spectrometry: progress in developing clinical diagnostic assays for tissues, biofluids, and cells. Clin Lab Med. (2021) 41:247–66. doi: 10.1016/j.cll.2021.03.005 PMC8862151 34020762

[66] Carter C Parker G Hankey K Farese A MacVittie T Kane M . Maldi-msi spatially maps n-glycan alterations to histologically distinct pulmonary pathologies following irradiation. Sci Rep. (2020) 10:11559. doi: 10.1038/s41598-020-68508-y 32665567 PMC7360629

[67] Wang I Song L Wang B Rezazadeh Kalebasty A Uchio E Zi X . Prostate cancer immunotherapy: a review of recent advancements with novel treatment methods and efficacy. Am J Clin Exp Urol. (2022) 10:210–33. PMC9428569 36051616

[68] Shakiba M Tuveson D . Macrophages and fibroblasts as regulators of the immune response in pancreatic cancer. Nat Immunol. (2025) 26:678–91. doi: 10.1038/s41590-025-02134-6 40263612 PMC12324146

[69] Xin S Wen S He P Zhao Y Zhao H . Density of tertiary lymphoid structures and their correlation with prognosis in non-small cell lung cancer. Front Immunol. (2024) 15:1423775. doi: 10.3389/fimmu.2024.1423775 39192984 PMC11347756

[70] Finkin S Yuan D Stein I Taniguchi K Weber A Unger K . Ectopic lymphoid structures function as microniches for tumor progenitor cells in hepatocellular carcinoma. Nat Immunol. (2015) 16:1235–44. doi: 10.1038/ni.3290 26502405 PMC4653079

[71] You X Koop K Weigert A . Heterogeneity of tertiary lymphoid structures in cancer. Front Immunol. (2023) 14:1286850. doi: 10.3389/fimmu.2023.1286850 38111571 PMC10725932

[72] Mai Z Lin Y Lin P Zhao X Cui L . Modulating extracellular matrix stiffness: a strategic approach to boost cancer immunotherapy. Cell Death Dis. (2024) 15:307. doi: 10.1038/s41419-024-06697-4 38693104 PMC11063215

[73] Moremen K Tiemeyer M Nairn A . Vertebrate protein glycosylation: diversity, synthesis and function. Nat Rev Mol Cell Biol. (2012) 13:448–62. doi: 10.1038/nrm3383 22722607 PMC3934011

[74] Han Y Lee S Lee J Jeong S Lee K Ahn S . Spatial transcriptomics reveal high t cell and monocyte status as predictive and prognostic markers in pancreatic cancer. J Transl Med. (2025) 23:576. doi: 10.1186/s12967-025-06599-9 40410886 PMC12102996

[75] Abdelbary M Nolz J . N-linked glycans: an underappreciated key determinant of t cell development, activation, and function. Immunometabolism (Cobham). (2023) 5:e00035. doi: 10.1097/in9.0000000000000035 38027254 PMC10662610

[76] Green R Stone E Tenno M Lehtonen E Farquhar M Marth JD . Mammalian n-glycan branching protects against innate immune self-recognition and inflammation in autoimmune disease pathogenesis. Immunity. (2007) 27:308–20. doi: 10.1016/j.immuni.2007.06.008 17681821

[77] He X Wang B Deng W Cao J Tan Z Li X . Impaired bisecting GlcNAc reprogrammed M1 polarization of macrophage. Cell Commun Signaling. (2024) 22:73. doi: 10.1186/s12964-023-01432-6 38279161 PMC10811823

[78] Li P Chen Z You S Xu Y Hao Z Liu D . Application of StrucGP in medical immunology: site-specific n-glycoproteomic analysis of macrophages. Front Med. (2023) 17:304–16. doi: 10.1007/s11684-022-0964-8 36580234

[79] Mortales C-L Lee S-U Demetriou M . N-glycan branching is required for development of mature B cells. J Immunol. (2020) 205:630–6. doi: 10.4049/jimmunol.2000101 32591389 PMC7417207

[80] Nie H Saini P Miyamoto T Liao L Zielinski R Liu H . Targeting branched n-glycans and fucosylation sensitizes ovarian tumors to immune checkpoint blockade. Nat Commun. (2024) 15:2853. doi: 10.1038/s41467-024-47069-y 38565883 PMC10987604

[81] Silva M Fernandes Â Oliveira M Resende C Correia A de-Freitas-Junior J . Glycans as immune checkpoints: removal of branched n-glycans enhances immune recognition preventing cancer progression. Cancer Immunol Res. (2020) 8:1407–25. doi: 10.1158/2326-6066.cir-20-0264 32933968

[82] Chakhachiro Z Saroufim M Safadi B Attieh M Assaf N Shamseddine G . Plasma cells and lymphoid aggregates in sleeve gastrectomy specimens: normal or gastritis? Med (Baltimore). (2020) 99:e18926. doi: 10.1097/md.0000000000018926 32028400 PMC7015648

[83] Berthe J Poudel P Segerer F Jennings E Ng F Surace M . Exploring the impact of tertiary lymphoid structures maturity in NSCLC: insights from TLS scoring. Front Immunol. (2024) 15:1422206. doi: 10.3389/fimmu.2024.1422206 39376565 PMC11457083

[84] Abdel Rahman A Ryczko M Pawling J Dennis J . Probing the hexosamine biosynthetic pathway in human tumor cells by multitargeted tandem mass spectrometry. ACS Chem Biol. (2013) 8:2053–62. doi: 10.1021/cb4004173 23875632

[85] Chandel N . Carbohydrate metabolism. Cold Spring Harb Perspect Biol. (2021) 13:a040568. doi: 10.1101/cshperspect.a040568 33397651 PMC7778149

[86] Conte F van Buuringen N Voermans N Lefeber D . Galactose in human metabolism, glycosylation and congenital metabolic diseases: time for a closer look. Biochim Biophys Acta (BBA) - Gen Subj. (2021) 1865:129898. doi: 10.1016/j.bbagen.2021.129898 33878388

