# Correction: Multi-modal spatial glycomic analysis of lymphoid aggregates in prostate and pancreatic cancer tissues

**DOI:** 10.3389/fonc.2026.2029746

**Published:** 2026-10-26

**Authors:** Kameisha V. Rashford, Caroline G. Kittrell, Lyndsay E. A. Young, Dessie Anne Nietert, Colin T. McDowell, Grace Grimsley, Blake E. Sells, Kaitlyn Bejar, Anand S. Mehta, Liang-I Kang, Peggi M. Angel, Jennifer D. Wu, David G. DeNardo, Robin J. Leach, Joseph E. Ippolito, Richard R. Drake

**Affiliations:** 1 Department of Pharmacology and Immunology and The Hollings Cancer Center, Medical University of South Carolina, Charleston, SC, United States; 2 Division of Oncology, Washington University in Saint Louis School of Medicine, Saint Louis, MO, United States; 3 Department of Cell Systems and Anatomy, University of Texas Health Science Center, San Antonio, TX, United States; 4 Department of Pathology and Immunology, Washington University in Saint Louis School of Medicine, Saint Louis, MO, United States; 5 Department of Urology, Feinberg School of Medicine, Northwestern University, Chicago, IL, United States; 6 Department of Radiology, Washington University in Saint Louis School of Medicine, Saint Louis, MO, United States

**Keywords:** lymphoid aggregates, MALDI-IHC, MALDI-MSI imaging, N-glycosylation, pancreatic adenocarcinoma, prostate adenocarcinoma, spatial biology, tumor microenvironment (TME)

There was a mistake in the caption of **Figure 2** as published. N-glycans co-localized with lymphoid aggregates. Representative spatial distribution images showing the most frequently detected N-glycan co-localized with LA in (A) PCa13 and (B) PDAC17 tissues. Frequency distributions of highly correlated N-glycans across LA in (C) prostate cancer (PCa) and (D) pancreatic ductal adenocarcinoma (PDAC) tissues. Frequency distributions of highly correlated N-glycans across LA in (A) prostate cancer (PCa) and (B) pancreatic ductal adenocarcinoma (PDAC) tissues. Following Pearson correlation analysis, the top 25 positively correlated N-glycans were identified within each tissue section, and the 15 most frequently recurring glycans are shown for each tumor type. Representative spatial distribution images showing the most frequently detected N-glycan colocalized with LA in (C) PCa13 and (D) PDAC17 tissues.

**Figure 2:**
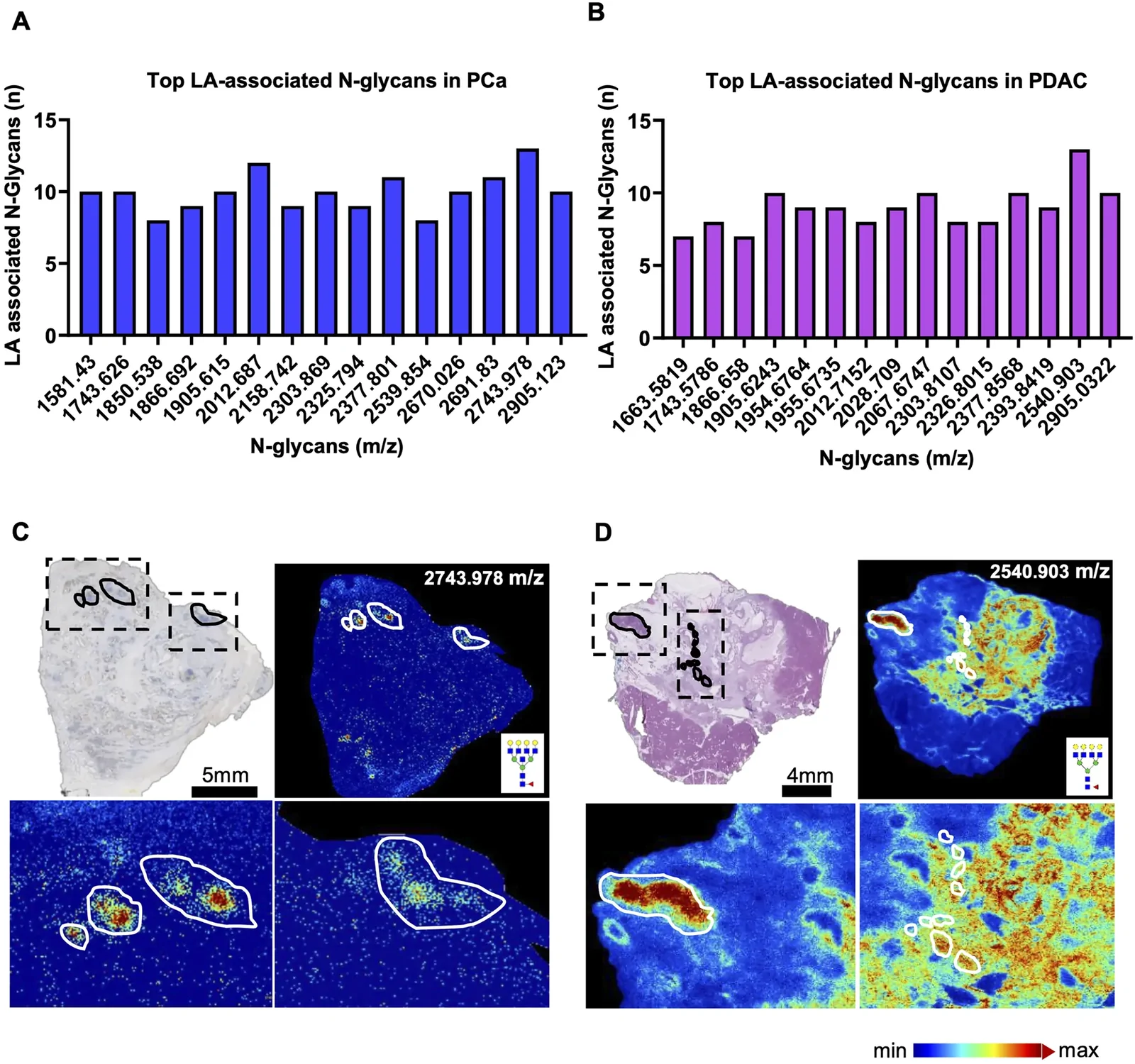
N-glycans co-localized with lymphoid aggregates. The 15 most recurrent N-glycans among the top 25 positive correlates per tissue are shown for **(A)** PCa and **(B)** PDAC. Representative spatial distribution images showing the most frequently detected N-glycan colocalized with LA in **(C)** PCa13 and **(D)** PDAC17 tissues.

The corrected caption of **Figure 2** appears below.

“N-glycans co-localized with lymphoid aggregates. The 15 most recurrent N-glycans among the top 25 positive correlates per tissue are shown for (A) PCa and (B) PDAC. Representative spatial distribution images showing the most frequently detected N-glycan colocalized with LA in (C) PCa13 and (D) PDAC17 tissues.”

The original version of this article has been updated.

